# Major Depressive Disorder with Catatonia: A Phenotype Related to Autistic Traits and High Suicidality

**DOI:** 10.3390/jcm13164796

**Published:** 2024-08-14

**Authors:** Barbara Carpita, Giulia Amatori, Ivan Mirko Cremone, Chiara Bonelli, Benedetta Nardi, Gabriele Massimetti, Stefano Pini, Liliana Dell’Osso

**Affiliations:** Department of Clinical and Experimental Medicine, Section of Psychiatry, University of Pisa, 67 Via Roma, 56126 Pisa, Italy; barbara.carpita1986@gmail.com (B.C.); ivan.cremone@gmail.com (I.M.C.); chiarabonelli.95@hotmail.it (C.B.); benedetta.nardi@live.it (B.N.); gabriele.massimetti@unipi.it (G.M.); stefano.pini@unipi.it (S.P.); liliana.dellosso@gmail.com (L.D.)

**Keywords:** major depressive disorder, catatonia, autism spectrum disorder, autistic traits, suicidality

## Abstract

**Background**: Major Depressive Disorder (MDD) represents a significant global health concern, often complicated by comorbidities such as catatonia and autism spectrum disorder (ASD). Recognizing the interplay among these conditions and their impact on suicidal tendencies is crucial for effective clinical management. **Methods**: A total sample of 147 subjects with MDD was divided into Significant Catatonia (SC) and Non-Significant Catatonia (NSC) groups based on Catatonia Spectrum (CS) scores. Participants were evaluated through the Structured Clinical Interview for DSM-5, Research Version (SCID-5-RV), the Adult Autism Subtreshold Spectrum (AdAS Spectrum), and the Mood Spectrum—Self Report questionnaires. Statistical analyses included Mann–Whitney U test, Chi-square test, logistic regression analyses, and a decision tree model. **Results**: The SC group exhibited higher CS, AdAS Spectrum, and MOODS-SR total and domain scores compared to the NSC group. Individuals with significant autistic traits were over-represented in the SC group, as well as participants with higher suicidality, suicidal ideation, and a history of suicide attempts. The total AdAS Spectrum and MOOD-SR score, the AdAS domain “Hyper-hypo reactivity to sensory input”, and the “Cognitive depressive” MOOD-SR domain were predictive of belonging to the SC group. Suicidality levels appeared to be higher in clinically significant ASD, intermediate in subjects with autistic traits (AT), and low in the absence of AT. **Conclusions**: the study suggests the existence of a specific phenotype of MDD associated with catatonia, characterized by elevated autistic traits and suicide risk.

## 1. Introduction

Major Depressive Disorder (MDD) represents a significant global health concern, currently ranking as the third leading cause of the burden of disease worldwide. According to the World Health Organization (WHO), projections suggest that by 2030, MDD will ascend to the forefront of this [1]. The diagnosis of MDD entails persistent low mood, anhedonia, feelings of guilt or worthlessness, and a range of other symptoms outlined in the Diagnostic and Statistical Manual of Mental Disorders, 5th Edition—Text Revision [2] (DSM-5-TR) Notably, MDD exhibits a higher prevalence in women than in men, affecting approximately 12% of the population over a lifetime [3]. This mental disorder not only impairs daily functioning but also detrimentally impacts interpersonal relationships, significantly diminishing quality of life. Moreover, individuals with MDD face an increased risk of developing comorbid mental disorders and, in this regard, catatonia represents one of the most severe potential comorbidities. Catatonia, a psychomotor syndrome associated with various psychiatric and medical conditions [2], poses a considerable threat to life, often necessitating urgent inpatient treatment [4]. Recognizing the presence of catatonia in individuals with MDD may not be so simple, however, especially in cases where akinetic manifestations predominate in the clinical presentation of catatonia. Indeed, one of the cardinal symptoms of catatonia, psychomotor retardation, shares similarities with depressive symptoms, particularly melancholia. This overlap raises questions about whether psychomotor symptoms in depression represent a milder form of catatonia [5]. Recognizing catatonic features, such as mutism and negativism, in patients with severe MDD is crucial, as treatment approaches differ from those for typical MDD. Furthermore, both depression and catatonia are closely linked to an increased risk of suicide. Studies have consistently demonstrated an association between MDD and suicidal behavior, with suicide risk rates reaching around 15% [6]. Recent research has also highlighted how elevated catatonic symptoms in psychiatric patients, including those with MDD, correlate with heightened suicidal tendencies [6]. In exploring the synergistic relationship between depression and catatonia, it is also pertinent to consider the potential role of the autism spectrum. Indeed, individuals with autism spectrum disorder (ASD) and autistic traits are characterized by a high rate of comorbidity with other mental disorders [7], such that it leads to the hypothesis in the literature of a neurodevelopmental basis for the various manifestations of psychiatric illnesses [8,9,10]. They are at a notably high risk of experiencing both MDD and catatonia throughout their lives, as well as self-injurious behaviors as a complication or manifestation of both mental disorders [11]. From a dimensional perspective, recent studies have hypothesized that alterations in neurodevelopment towards autism may serve as a vulnerability substrate for the development of a psychopathological trajectory leading to mood disorders and often culminating in severe catatonic manifestations [12]. Comorbidity with mood disorders would not only concern overt ASD but also subthreshold autistic traits [13,14] and, in the relationship between autistic traits and catatonic manifestations, the spectrum of mood disorders may play a mediating role [15]. In this regard, a study conducted on a large sample of individuals with MDD showed that the presence of autistic traits was associated with a higher risk and lethality of suicide attempts [16]. A recent systematic review has shown that in young individuals with autism (under 25 years old), the prevalence of suicide risk is significantly higher both compared to adult individuals with ASD and healthy controls of the same age [17]. In terms of a psychopathological trajectory originating from the autism spectrum, this finding is particularly interesting, highlighting how suicidal risk in autistic patients is already present at a young age, likely due to characteristics inherent to the disorder, and not just because of the frequent comorbidities that tend to occur throughout their lives. More specifically, among the different domains of the autistic spectrum, the dimension of mental rumination seems to be more strongly associated with suicide risk [18].

This could be interpreted considering the high predisposition of autistic individuals to experience traumatic events and develop post-traumatic symptoms, correlated with mental rumination [19] and suicidality [20]. Closing the circle, a recent study has shown that mental rumination and post-traumatic symptomatology partially mediate the significant relationship between autistic traits and mood spectrum [21].

Given the current understanding in the field and the limited research concerning the interplay among MDD, catatonia, and autism spectrum, this study aimed to examine the presence and severity of autism spectrum symptoms, mood symptoms, and suicidal tendencies in individuals diagnosed with Major Depressive Disorder (MDD), with and without significant catatonic features.

Considering what has been discussed so far, we expect that individuals with MDD who exhibit significant catatonic features will also show elevated autism spectrum symptoms. Moreover, we expect to observe higher suicidal tendencies in these individuals compared to those with MDD without catatonic features.

## 2. Materials and Methods

### 2.1. Study Sample and Procedures

Data were collected at the Department of Psychiatry of the Azienda Ospedaliero Universitaria Pisana (AOUP) between November 2021 and January 2022. The study involved 147 individuals diagnosed with Major Depressive Disorder (MDD), who were categorized into two groups based on their CS total score:

1. Significant Catatonia (SC) group, exhibiting a CS score of ≥30;
2. Non-Significant Catatonia (NSC) group, defined by a CS score <30.

Exclusion criteria encompassed individuals under 18 years old, those with language or intellectual impairments hindering assessment completion, individuals with mental disabilities, poor cooperation skills, or ongoing psychotic symptoms. All participants were aged between 18 and 60 years old and provided written informed consent. Diagnoses of Major Depressive Disorder were confirmed using the Structured Clinical Interview for DSM-5, Research Version [22]. The study adhered to the principles outlined in the Declaration of Helsinki. Approval for recruitment and assessment procedures was obtained from the Ethics Committee of the Azienda Ospedaliero Universitaria of Pisa on 27 October 2021 (Approval ID: 20608). Eligible participants provided informed consent after receiving detailed information about the study and having the opportunity to seek clarification. Participation was voluntary, and participants did not receive compensation in accordance with Italian legislation.

### 2.2. Measures

#### 2.2.1. Catatonia Spectrum (CS)

The CS questionnaire serves as a self-report tool devised to investigate various facets of the catatonic spectrum, encompassing core, subthreshold, atypical, and partial symptoms experienced over an individual’s lifetime. It comprises eight domains, each addressing distinct aspects: “Psychomotor activity (Stupor)”, “Verbal response (Mutism)”, “Repetitive movements (Stereotypes)”, “Artificial expressions and actions (Mannerisms)”, “Oppositivity or poor response to stimuli (Negativism)”, “Oppositivity or poor response to stimuli (Negativism)”, “Response to instructions given from outside (Automatic obedience)”, “Automatisms”, and “Impulsivity”. With a total of 74 items, respondents provide binary responses (“Yes” or “No”) to each question. In the validation study, the CS questionnaire exhibited notable internal consistency and test–retest reliability, alongside robust convergent validity, when compared to other dimensional assessments of catatonia, such as the Bush–Francis Catatonia Rating Scale and the Bush–Francis Catatonia Screening Instrument [23]. Recent research indicated that the optimal threshold for identifying catatonia through the CS questionnaire was a score of 30, demonstrating favorable levels of specificity (0.72) and sensitivity (1.00) [24].

#### 2.2.2. Adult Autism Subthreshold Spectrum (AdAS Spectrum)

The AdAS Spectrum questionnaire aims to assess not only clinically significant ASD, but also the broader range of subthreshold autism in individuals with normal intelligence and without language impairment throughout their lifespan. It enables the evaluation of diverse clinical and non-clinical traits, encompassing typical and atypical manifestations, including certain gender-specific features. The questionnaire comprises dichotomous questions organized into seven domains: “Childhood/adolescence”, “Verbal communication”, “Non-verbal communication”, “Empathy”, “Inflexibility and adherence to routine”, “Restricted interests and rumination”, and “Hyper-hypo reactivity to sensory input”. In the validation study [25], the AdAS Spectrum questionnaire exhibited excellent reliability and strong convergent validity with other established scales in this field, such as the Autism-Spectrum Quotient Test and the Ritvo Autism and Asperger Diagnostic Scale 14-item version. A score equal to or greater than 70 indicates the presence of possible clinical ASD, while a score ranging from 69 to 43 suggests significant autistic traits (ATs) or subthreshold autism spectrum [26].

#### 2.2.3. Mood Spectrum Self-Report (MOOD-SR)

The MOODS-SR questionnaire [27] was designed to evaluate a wide range of mood symptoms, including suicidal thoughts and behaviors, as well as temperamental traits throughout an individual’s life. As a dimensional tool, it aims to detect even subtle and subthreshold presentations, which may manifest with prodromal, residual, or atypical clinical features. This instrument categorizes items into three domains related to manic symptoms and three domains related to depressive symptoms, exploring “mood”, “energy”, and “cognition”, alongside a domain focusing on rhythmicity and vegetative functions. The latter domain examines alterations in circadian rhythms and vegetative functions, including changes in energy levels, physical well-being, mental and physical efficiency influenced by weather and season, appetite, sleep, and sexual activities, spanning a total of 29 items. The rhythmicity subdomain comprises six items investigating mood, energy, interests, and efficiency fluctuations throughout the year or even within a day, influenced by factors such as weather, season, menstrual cycle phase, or disruptions in circadian rhythms. The vegetative function subdomains encompass sleep (12 items assessing insomnia, sleepiness, reduced need for sleep, sleep changes due to external stimuli, season, jet lag, and menstrual cycle), weight and appetite (4 items addressing changes in taste, appetite, weight, and carbohydrate cravings), sexual functions (5 items examining reduced sexual interest, difficulties with sexual stimulation and orgasm, increased sexual interest, and promiscuity tendencies), and physical symptoms (5 items concerning headache, dry mouth, constipation, nausea, and other gastrointestinal issues). Furthermore, the following six MOOD-SR items were combined to assess the suicidality: “thought that life is not worth living” (item 102); “wished he/she would not wake up in the morning, or he/she would die in an accident or from something like a heart attack or a stroke” (item 103); “wanted to die or hurt him/herself ” (item 104); “wanted to die and had a specific plan to hurt or kill him/herself ” (item 105); “actually committed a suicide attempt” (item 106); and “commita suicide attempt that required medical attention” (item 107). The first four items represent a novel dichotomous variable called “suicidal ideation”, which results from the endorsement of at least one item among those mentioned above. A second new dichotomous variable named “suicidal attempts” was derived from the endorsement of at least one out of the last two items (106 and/or 107). The Mood Spectrum Self-Report–Lifetime Version, which is the self-report format of the Structured Clinical Interview for Mood Spectrum (SCI-MOODS), demonstrated robust intraclass correlation coefficients (ranging from 0.88 to 0.97) when compared to the interview-based SCI-MOODS format [27].

#### 2.2.4. Statistical Analyses

All statistical analyses were conducted using the Statistical Package for Social Science, version 25.0 (SPSS Inc., IBM Corp., Armonk, NY, USA). Comparisons between questionnaire scores among the sample groups were performed trough the Mann–Whitney U Test. A *p* value < 0.05 was considered statistically significant. Chi-square tests were used in order to compare sex composition among groups and to test the relationships among the ASD, AT, and non-AT groups. Logistic regression analyses were used in order to identify the best predictor for the presence of clinically significant catatonic symptoms. Finally, a decision tree model was performed for identifying which variables among the presence/absence of catatonia and presence/absence of AT or clinically significant ASD symptoms best predicted suicidality total scores. This analysis allows examining the interactions among variables, building a decision tree model that graphically represents findings as an inverted tree. Starting with a root node with all the cases included, the tree model grows at each step by choosing the independent variable with the higher interaction with the dependent variable. When no difference is reported with respect to the dependent variable, the categories defined by predictors are automatically merged.

## 3. Results

### 3.1. Sample Composition

The total sample included 147 participants, including 54 (36.7%) males and 93 females (63.3%). The NSC group was composed of 75 (51.0%) participants, 50 (66.7%) females and 25 (33.3%) males, with a mean age of 46.0 years (±12.351), while the SC group included 72 (49.0%) individuals, 43 (59.7%) females and 29 (40.3%) males, with mean age of 44.424 years (±12.557). The two groups did not significantly differ from each other in terms of age (*p* = 0.686; t = 1.099) and sex (*p* = 0.383; X^2^ = 0.762). A summary of data is available in Table 1.

### 3.2. Comparisons between CS Total and Domain Scores among the CS Groups

The CS total and domain scores obtained by the SC group were always significantly higher than the respective scores observed in the NSC group. The data are reported in Table 2.

### 3.3. Comparisons between AdAS Spectrum Total and Domain Scores among the CS Groups

As for the CS, the AdAS Spectrum total and domain scores were found to be significantly higher in the SC compared to the NSC group. The results are reported in Table 3.

### 3.4. Distribution of Autistic Traits within Groups

Based on the presence of autistic symptoms, determined from the total AdAS Spectrum score, the total sample was divided into three subgroups:

1. Participants with possible clinical symptoms of ASD (ASD group), composed of 40 subjects (27.2%);
2. Participants with subthreshold autistic traits (AT group), composed of 57 subjects (38.8%);
3. Participants without autistic traits (non-AT group), composed of 50 subjects (34.0%).

The relationships among the ASD, AT, and non-AT groups and the two catatonia groups were tested using the Chi-square test, showing a significantly higher proportion of subjects with ASD in the NSC group and of subjects with non-AT in the NSC group (*p* < 0.01; X^2^ = 58.002).

Table 4 shows the size of each CS group for each AdAS category, the percentage of subjects belonging to each AdAS category within the CS group (% within CS group), and the percentage of subjects belonging to each CS group within the AdAS category (% within AdAS group).

The distribution of each CS group within the AdAS categories was as follows:

1. NSC group: 60.0% in the non-AT group, 34.7% in the AT group, and 5.3% in the ASD group;
2. SC group: 6.9 % in the non-AT group, 43.1% in the AT group, and 50.0% in the ASD group.

So, considering the composition of each of the three AdAS categories, it was possible to observe the following:

1. The non-AT group consisted of 90.0% of subjects from the NSC group and 10.0% of individuals from the SC group.
2. The AT group consisted of 45.6% of participants of the NSC group and 54.4% of the SC group.
3. The ASD group consisted of 10.0% of subjects belonging to the NSC group and 90.0% to the SC group.

### 3.5. Comparisons between MOODS-SR Total and Domain Scores among the Groups

Similar to what was observed in the analysis of the two previous questionnaires, the MOOD-SR total and domain scores were found to be significantly higher in the SC than in the NSC group. The respective data can be consulted in Table 5.

### 3.6. Suicidality among the Two CS Groups

By dividing the total sample based on the presence of “Suicidality,” observed from satisfaction with the corresponding item, into two groups—”Non-Suicidality” and “Suicidality”—a significant difference was noted (*p* < 0.01; X^2^ = 20.241); in particular, most individuals (62.5%) in the first group belonged to the SC group, while the majority of “suicidality” subjects (76.5%) belonged to the NSC group. Additional details can be found in Table 6.

The total sample was further divided, based on the satisfaction of the “suicidal ideation” item, into two groups—“Suicidal Ideation” (satisfaction with the corresponding item), with 73.8% of subjects belonging to the SC group, and “Non-Suicidal Ideation” (lack of satisfaction with the corresponding item), with 77.4% of participants in the NSC group—with a significant difference in the proportion between group (*p* < 0.01; X^2^ = 23.008). Similarly, based on the satisfaction of the “Suicidal Attempts” item, two groups were identified—“Suicidal Attempts” (satisfaction with the corresponding item), with 69.1% of subjects in the SC group, and “Non-Suicidal Attempt” (lack of satisfaction with the corresponding item), in which 63.0% of subjects were classified in the NSC group—reporting a significant difference in the distribution between groups (*p* < 0.01; X^2^ = 14.224). Further details are available by referring to Table 7 and Table 8, respectively.

### 3.7. Regression Analyses

In order to deepen our analysis, logistic regressions were conducted using the CS groups (NSC and SC) as dependent variables.

In the first logistic regression, total AdAS Spectrum and MOOD-SR scores were used as independent variables, showing that they were both predictive of belonging to the SC group (Table 9).

In the second logistic regression, the AdAS Spectrum domains were used as independent variables and only the “Hyper-hypo reactivity to sensory input” domain appeared to be predictive of belonging to the SC group (Table 9).

In the third logistic regression, MOOD-SR domains “Mood Depressive”, “Mood Manic”, “Energy Depressive”, “Energy Manic”, “Cognition Depressive”, “Cognition Manic”, “Rhytmicity”, and “Suicidality” were used as independent variables, showing a predictive role of the “Cognition depressive” domain for membership in SC group (Table 9).

### 3.8. Decision Tree Model

**Figure 1:**
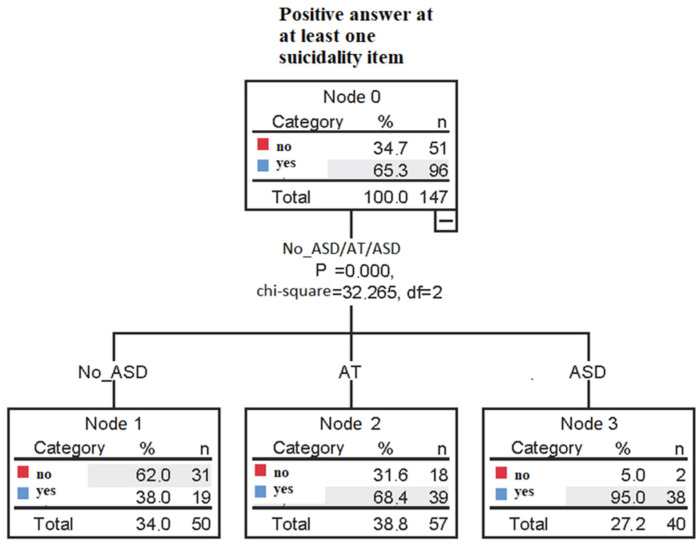
Decision tree model using presence/absence of catatonia and AT or ASD as independent variables and presence/absence of positive endorsement to at least one suicidality item as the dependent variable.

The decision tree model, built with the presence/absence of catatonia and presence/absence of AT or clinically significant ASD symptoms as independent variables and presence/absence of positive endorsement to at least one suicidality item as the dependent variable, showed in the first step a higher rate of suicidality among subjects with clinically significant ASD symptoms and an intermediate rate in AT subjects, while the lowest rates were reported in non-AT subjects. No effect was found for the presence/absence of catatonia (see Figure 1).

## 4. Discussion

As highlighted by the results, both the CS total and domain scores were significantly higher in the SC group compared to the NSC group. This finding confirms the higher CS scores, on all domains, in the SC group. It is important to note that the use of CS as an assessment tool allowed for a thorough evaluation of catatonic symptoms in this population. However, it is necessary to consider that higher CS scores in the SC group compared to the NSC group were expected, given that the groups were divided precisely based on these scores.

The observation of significantly higher scores on the autism spectrum questionnaire in the SC compared to NSC group aligns with the existing literature on the high comorbidity between catatonia and ASD [28] and between MDD and ASD [29]. Cases of catatonia are frequently observed in adolescents with psychiatric diagnoses and children with neurodevelopmental disorders, particularly ASD [30]. For instance, 18% of adolescents admitted to a specialized psychiatric ward with pervasive developmental disorder, psychosis, disruptive behavior disorder, and intellectual disability exhibited pronounced symptoms of catatonia [31]. Furthermore, in two prevalence studies [32,33], a diagnosis of catatonia was made in 12–17% of a large sample of adolescents and young adults with ASD. Moreover, there is significant clinical overlap between autism and catatonia, including mutism, echolalia, stereotyped movements, repetitive behaviors, negativism, and excitement. Catatonia can slowly evolve during autism, often preceded by isolated symptoms and a slow deterioration in functioning [34].

Unfortunately, catatonia occurring over the lifespan in autistic patients may go unnoticed or be attributed to other mental disorders. For example, psychomotor slowing may be mistaken for melancholic depression, or catatonia may arise as a complication of Major Depressive Disorder. The lifetime prevalence for adults with ASD has been estimated between 23 and 37% for depressive disorders, with peaks among individuals without intellectual disability. These conditions may imply risks in individuals with ASD, especially considering that prevalence rates for suicidal ideation range from 11 to 66%, and suicidal attempts range from 1 to 35% in ASD [35]. Moreover, Hirvikoski et al. reported that 0.31% of premature deaths in ASD were due to suicide [36]. On the other hand, subjects with full-blown ASD without intellectual or language impairment may remain under-detected, reaching clinical attention only when developing other psychiatric disorders in comorbidity such as mood or anxiety disorders [15,37,38]. In this cases, the unrecognized ASD, or the presence of subthreshold AT, typically exerts a detrimental effect on the clinical picture [37].

In this regard, the results of this study also highlight that the majority of subjects with high suicidal tendencies, suicidal ideation, and at least one suicide attempt in their clinical history were located in the MDD group with significant catatonic symptoms. This finding sheds light on how the coexistence of MDD and catatonic symptoms can generate extremely severe clinical pictures, potentially complicated by the presence of high autistic traits, which, as previously reported, are also associated with high suicidality.

The logistic regressions conducted further deepened our understanding of factors associated with the presence of significant catatonic symptoms in subjects with MDD. For instance, it emerged that both total scores on the AdAS Spectrum and the MOOD-SR were predictive of belonging to the SC group, suggesting a relevant role of significant autistic traits and mood symptoms in differentiating patients with and without Significant Catatonia. Furthermore, in the analysis of AdAS Spectrum domains, the “Hyper-hypo reactivity to sensory input” domain was found to be predictive of belonging to the SC group. This result could be discussed in light of the hypothesis of catatonia as a pathological response to fear and trauma [39]. The role of trauma in influencing a vulnerable individual to develop catatonic symptoms was explored in a recent study, in which a meaningful correlation was found between the Bush–Francis Catatonia Scale and the Adverse Childhood Experience questionnaire [40]. Based on the same study, there appear to be two constructs of conditioning that predispose a subject to catatonic symptoms in response to traumatic events: external conditioning (e.g., the external environment) and internal conditioning (e.g., flashbacks or nightmares after a traumatic experience). In this context, the hypersensitivity typical of autistic individuals could be responsible for an increased intensity of post-traumatic re-experiencing, which is known to be associated with an altered processing of sensations from the external and internal world [41,42]. This perspective would be in agreement with the so-called “Intense World Theory” according to which individuals with autism are characterized by hyper-perception, hyper-attention, hyper-memory, and hyper-emotionality. Furthermore, within the framework of a psychopathological trajectory originating from the autistic spectrum and directed, through the intermediation of trauma, towards catatonic manifestations, hypersensitivity to sensory stimuli could render the post-traumatic re-experiencing more vivid and painful, facilitating illness progression.

Furthermore, the results from the decision tree model show how the presence or absence of ASD or autistic traits appear to be more influential in predicting suicide risk compared to the presence or absence of catatonia. This finding would seem to support the role of the autism spectrum as the “primum movens” in influencing suicidality within the psychopathological trajectory.

As for the MOOD-SR, the “Depressive cognitions” domain was found to increase the risk of belonging to the SC group. This finding could be explained by considering the extreme heterogeneity of the diagnostic criteria of the DSM for the diagnosis of MDD, while the individual with MDD and catatonia could represent a much less heterogeneous and more “severe” phenotype of depression, closer to the original conceptualization of “melancholia” [43], associated with intense depressive cognitions.

In the current landscape of scientific literature, we believe that the originality of this work lies in being the first study to analyze the correlation between MDD, catatonia, autism, and suicidality. Additionally, it has opened the hypothesis of a specific subtype of MDD characterized by autistic traits, catatonic features, and high suicidality. This subtype can be further explored in future clinical and biological studies to assess its potential proximity to neurodevelopmental disorders and its position within the neurodevelopmental continuum [44].

Several limitations need consideration: the sample size was relatively small, the questionnaires relied on self-reporting, potentially causing symptom overestimation or underestimation by patients, and the study’s cross-sectional design limited the exploration of temporal and causal relationships among the investigated variables.

## 5. Conclusions

In conclusion, this study reveals a significant convergence of evidence regarding the interconnection between MDD, catatonia, AT, and suicidality. This complex inter-relation highlights the need for an integrate perspective in the diagnostic and therapeutic approach to such mental disorders. The findings indicate that a better understanding of phenotypic overlaps can contribute to more accurate identification of at-risk patients and more targeted clinical management. However, further research is needed to more clearly delineate the underlying pathophysiological pathways and to develop personalized therapeutic approaches.

## Figures and Tables

**Table 1:** Frequency and demographic characteristics of the sample.

CS Groups	Frequency (Percent)	Mean Age (Standard Deviation)	Sex (Percent, %)
Non-Significant Catatonia (NSC)	75 (51.0%)	46.00 (12.351)	Females = 50 (66.7%) Males= 25 (33.3%)
Significant Catatonia (SC)	72 (49.0%)	41.00 (12.69)	Females = 43 (59.7%) Males= 29 (40.3%)

**Table 2:** Comparisons between CS total and domain scores among the CS groups (NSC and SC).

CS Domains	NSC Group Mean ± sd (Mean Rank)	SC Group Mean ± sd (Mean Rank)	*p*	U	W
Psychomotor activity (Stupor)	5.48 ± 3.31 (42.31)	11.51 ± 2.17 (107.01)	<0.01	5076.500	7704.500
Verbal response (Mutism)	2.29 ± 2.13 (46.43)	6.07 ± 2.06 (102.72)	<0.01	4768.00	7396.00
Repetitive movements (Stereotypes)	0.97 ± 1.85 (48.01)	3.22 ± 1.71 (101.08)	<0.01	4649.50	7277.500
Artificial expressions and actions (Mannerisms)	0.28 ± 0.69 (48.84)	2.29 ± 2.03 (100.21)	<0.01	4587.00	7215.00
Oppositivity or poor response to stimuli (Negativism)	1.47 ± 1.57 (45.47)	4.59 ± 1.76 (103.72)	<0.01	4839.500	7467.500
Response to instructions given from outside (Automatic obedience)	1.96 ± 1.55 (52.58)	3.93 ± 1.67 (69.63)	<0.01	4329.00	6957.00
Automatisms	2.06 ± 1.76 (46.31)	6.09 ± 2.61 (102.85)	<0.01	4777.00	7405.00
Impulsivity	2.19 ± 1.85 (48.11)	6.44 ± 3.36 (100.97)	<0.01	4642.00	7270.00
Total Score	16.70 ± 8.16 (38.00)	16.70 ± 8.16 (111.50)	<0.01	5400.00	8028.00

**Table 3:** Comparisons between AdAS Spectrum total and domain scores among the CS groups (NSC and SC).

AdAS Spectrum Domains	NSC Group Mean ± sd (Mean Rank)	SC Group Mean ± sd (Mean Rank)	*p*	U	W
Childhood/adolescence	5.28 ± 3.94 (54.79)	9.75 ± 4.93 (94.01)	<0.01	4140.500	6768.500
Verbal Communication	3.95 ± 3.41 (50.85)	8.01 ± 3.21 (98.11)	<0.01	4436.00	7064.00
Non-Verbal Communication	6.48 ± 4.83 (51.05)	12.50 ± 4.97 (97.91)	<0.01	4421.50	7049.500
Empathy	2.71 ± 2.63 (59.44)	4.90 ± 3.19 (89.17)	<0.01	3792.00	6420.00
Inflexibility and adherence to routine	9.43 ± 6.89 (49.49)	19.60 ± 8.42 (99.53)	<0.01	4538.500	7166.500
Restricted interests and rumination	5.97 ± 4.51 (51.04)	11.64 ± 4.26 (97.92)	<0.01	4422.00	7050.00
Hyper-hypo reactivity to sensory input	2.73 ± 2.94 (48.47)	7.62 ± 4.04 (99.56)	<0.01	4540.00	7168.00
Total score	35.54 ± 24.30 (47.72)	74.03 ± 27.01 (101.38)	<0.01	4671.00	7299.00

**Table 4:** Distribution of the two CS groups (NSC and SC) among the three autistic severity groups (non-AT, AT, and ASD).

AdAS Groups	Non-AT (*n*, %)	AT (*n*, %)	ASD (*n*, %)	Total (*n*, %)
NSC				
*n* *	45_a_ *	26_b_ *	4_c_ *	75
% within diagnosis	60.0%	34.7%	5.3%	100.0%
% within AdAS group	90.0%	45.6%	10.0%	51.0%
SC				
*n* *	5_a_ *	31_b_ *	36_c_ *	72
% within diagnosis	6.9%	43.1%	50.0%	100.0%
% within AdAS group	10.0%	54.4%	90.0%	51.0%

*p* < 0.01; X^2^ = 58.002. * Each subscript letter denotes a subset of CS groups whose column proportions do not differ significantly from each other at the 0.05 level.

**Table 5:** Comparisons between MOOD-SR total and domain scores among the CS groups (NSC and SC).

MOOD-SR Domains	NSC Group Mean ± sd (Mean Rank)	SC Group Mean ± sd (Mean Rank)	*p*	U	W
Mood Depressive	12.28 ± 6.54 (59.62)	17.72 ± 5.34 (91.79)	<0.01	3981.00	6609.00
Mood Manic	7.17 ± 5.36 (56.85)	11.90 ± 5.91 (91.87)	<0.01	3986.500	6614.500
Energy Depressive	3.44 ± 2.72 (55.28)	6.08 ± 2.70 (93.50)	<0.01	4104.00	6732.00
Energy Manic	3.32 ± 2.76 (58.35)	5.57 ± 3.16 (90.11)	<0.01	3860.00	6488.00
Cognition Depressive	8.64 ± 5.74 (52.61)	15.78 ± 6.15 (96.28)	<0.01	4304.00	6932.00
Cognition Manic	4.17 ± 4.18 (59.99)	7.85 ± 5.83 (88.59)	<0.01	3750.00	6378.500
Rhythmicity and vegetative functions	10.21 ± 6.00 (55.07)	15.72 ± 5.22 (93.72)	<0.01	4120.00	6748.00
Mood total	49.24 ± 25.96 (50.52)	80.62 ± 25.13 (98.46)	<0.01	4461.00	7089.00
Mood total—Depressive	24.36 ± 13.15 (52.05)	39.58 ± 12.00 (69.90)	<0.01	4348.500	6976.500
Mood total—Manic	14.67 ± 11.06 (55.35)	25.32 ± 12.56 (93.42)	<0.01	4089.500	6726.500
Rhythmicity	2.36 ± 1.56 (55.34)	3.86 ± 1.47 (93.44)	<0.01	4099.500	6272.500
Sleep	4.20 ± 2.96 (59.98)	6.19 ± 2.66 (88.60)	<0.01	3751.500	6379.500
Weight and appetite	1.25 ± 1.28 (59.07)	2.24 ± 1.23 (89.55)	<0.01	3819.500	6447.500
Sexual functions	1.49 ± 1.45 (67.25)	1.99 ± 1.51 (81.03)	<0.01	3206.500	5834.500
Physical Symptoms	1.11 ± 0.00 (59.20)	1.90 ± 1.04 (89.42)	<0.01	3810.00	6438.00
Suicidality	1.52 ± 1.96 (58.04)	3.18 ± 2.05 (90.63)	<0.01	3897.00	6525.00
Suicidal Ideation	1.16 ± 1.49 (58.31)	2.37 ± 1.49 (90.35)	<0.01	3877.00	6505.00
Suicidal Attempts	0.36 ± 0.71 (63.4)	0.81 ± 0.85 (85.01)	<0.01	3493.00	6121.00

**Table 6:** Distribution of the two CS groups (NSC and SC) among the two “suicidality” groups.

CS Groups	Non-Suicidality (*n*, %)	Suicidality (*n*, %)	Total (*n*, %)
NSC			
*n* *	39_a_	36_b_	75
% within diagnosis	52.0%	48.0%	100.0%
% within CS group	76.5%	37.5%	51.0%
SC			
*n* *	12_a_	60_b_	72
% within diagnosis	16.7%	83.3%	100.0%
% within CS group	23.5%	62.5%	49.0%

*p* < 0.01; X^2^ = 20.241. * Each subscript letter denotes a subset of “Suicidality” groups whose column proportions do not differ significantly from each other at the 0.05 level.

**Table 7:** Distribution of the two CS groups (NSC and SC) between the two “suicidal ideation” groups. Comparisons between CS total and domain scores among the CS groups (NSC and SC).

CS Groups	Non-Suicidal Ideation (*n*, %)	Suicidal Ideation (*n*, %)	Total (*n*, %)
NSC			
*n* *	41_a_	34_b_	75
% within diagnosis	54.7%	45.3%	100.0%
% within CS group	77.4%	36.2%	51.0%
SC			
*n* *	12_a_	60_b_	72
% within diagnosis	16.7%	83.3%	100.0%
% within CS group	22.6%	63.8%	49.0%

*p* < 0.01; X^2^ = 23.008. * Each subscript letter denotes a subset of “Suicidal Ideation” groups whose column proportions do not differ significantly from each other at the 0.05 level.

**Table 8:** Logistic regression using CS groups as dependent variables and the AdAS Spectrum total score and MOOD-SR total scores as independent variables.

Independent Variables	B(SE)	OR	*p*	CI (95%)
Constant	−4.154 (0.720)	0.016	<0.001	-
AdAS Spectrum total score	0.047 (0.011)	1.048	<0.001	1.026; 1.070
MOOD-SR total score	0.024 (0.009)	1.025	0.007	1.007; 1.043

Cox–Snell R square = 0.384; Nagelkerke R = 0.512; Overall percentage correct: 81.6%.

**Table 9:** Logistic regression using CS groups as dependent variables and AdAS Spectrum domain scores as independent variables.

	B	OR	*p*	
Independent Variables	B(SE)	OR	*p*	CI (95%)
Constant	−3.016 (0.595)	0.049	<0.001	-
Hyper-hypo reactivity to sensory input	0.183 (0.088)	1.201	0.038	1.010; 1.427

Cox–Snell R square = 0.368; Nagelkerke R = 0.490; Overall percentage correct: 76.9%.

## Data Availability

The data that support the findings of this study are available on request from the corresponding author. The data are not publicly available due to containing information that could compromise the privacy of the research participants.
